# Targeting Aurora B kinase with Tanshinone IIA suppresses tumor growth and overcomes radioresistance

**DOI:** 10.1038/s41419-021-03434-z

**Published:** 2021-02-04

**Authors:** Ming Li, Haidan Liu, Qin Zhao, Shuangze Han, Li Zhou, Wenbin Liu, Wei Li, Feng Gao

**Affiliations:** 1 Cell Transplantation and Gene Therapy Institute, The Third Xiangya Hospital, Central South University, Changsha, 410013 Hunan People’s Republic of China; 2 Changsha Stomatological Hospital, Changsha, 410004 Hunan People’s Republic of China; 3 School of Stomatology, Hunan University of Chinese Medicine, Changsha, 410208 Hunan People’s Republic of China; 4 Xiangya Stomatological Hospital & School of Stomatology, Central South University, Changsha, 410000 Hunan People’s Republic of China; 5 Clinical Center for Gene Diagnosis and Therapy, The Second Xiangya Hospital of Central South University, Changsha, 410011 Hunan People’s Republic of China; 6 Department of Radiology, The Third Xiangya Hospital of Central South University, Changsha, 410013 Hunan People’s Republic of China; 7 Department of Pathology, Xiangya Hospital, Changsha, 410008 Hunan People’s Republic of China; 8 Department of Pathology, Hunan Cancer Hospital, Changsha, 410013 Hunan People’s Republic of China; 9 Department of Ultrasonography, The Third Xiangya Hospital of Central South University, Changsha, 410013 Hunan People’s Republic of China

**Keywords:** Oral cancer, Cell signalling

## Abstract

Aurora B kinase is aberrantly overexpressed in various tumors and shown to be a promising target for anti-cancer therapy. In human oral squamous cell carcinoma (OSCC), the high protein level of Aurora B is required for maintaining of malignant phenotypes, including in vitro cell growth, colony formation, and in vivo tumor development. By molecular modeling screening of 74 commercially available natural products, we identified that Tanshinone IIA (Tan IIA), as a potential Aurora B kinase inhibitor. The in silico docking study indicates that Tan IIA docks into the ATP-binding pocket of Aurora B, which is further confirmed by in vitro kinase assay, ex vivo pull-down, and ATP competitive binding assay. Tan IIA exhibited a significant anti-tumor effect on OSCC cells both in vitro and in vivo, including reduction of Aurora B and histone H3 phosphorylation, induction of G2/M cell cycle arrest, increase the population of polyploid cells, and promotion of apoptosis. The in vivo mouse model revealed that Tan IIA delayed tumor growth of OSCC cells. Tan IIA alone or in combination with radiation overcame radioresistance in OSCC xenograft tumors. Taken together, our data indicate that Tan IIA is an Aurora B kinase inhibitor with therapeutic potentials for cancer treatment.

## Introduction

Oral squamous cell carcinoma (OSCC), a malignancy that arises from the epithelial mucosa of the oral cavity, is a public health problem worldwide. The incidence and mortality of OSCC have been increased over the past few decades, and over 95% of people with OSCC smoke tobacco, drink alcohol, and chew betel nut^1,2^. For most OSCC early cases, surgery is the initial treatment of choice. However, OSCC is frequently diagnosed at an advanced stage, and postoperative radiation or chemoradiation is needed if the disease has high-risk features. The distant metastasis remains the major reason to cause relapse and therapy failure, and the 5-year overall survival (OS) of OSCC patients was less than 50%^3–5^. Further elucidate the underlying mechanism of OSCC oncogenesis, and the discovery of critical drivers are the major challenges for OSCC treatment.

Unlimited cell growth is a hallmark of human cancer cells, and targeting cell cycle progression is considered a promising anti-tumor strategy for clinical treatment^6,7^. The Aurora kinases play crucial roles in orchestrating cell mitosis. In mammalian cells, the Aurora kinases comprise three family members, including Aurora A, B, and C, and exhibited distinct subcellular locations and functions during mitosis^8–10^. Aurora A is localized at the centrosome and mainly mediates the prometaphase events. As a chromosomal passenger protein, Aurora B is involved in directing metaphase–anaphase transition and is required for chromosome alignment, accurate segregation, and cytokinesis^10^. Suppression of Aurora B kinase activity results in cytokinesis failure and abnormal exit from mitosis, which leads to endoreduplication, polyploidy cells, and eventually apoptosis^8^. Aurora C is considered to have a similar subcellular location and redundant functions and substrates as Aurora B^10^. In OSCC, Aurora B expression was well correlated with cell proliferation, induction of multinuclear cells, and metastasis. Moreover, overexpression of Aurora B and upregulation of nuclear Aurora B immunolocalization indicate a worse prognosis^11,12^. This evidence suggests that Aurora B might be an interesting target for OSCC treatment.

In the present study, with a computational docking screening, we identified Tanshinone IIA (Tan IIA) as a novel Aurora B kinase inhibitor. We determined the in vitro and in vivo therapeutic effect of Tan IIA in OSCC cells and investigated the underlying molecular mechanism of action.

## Material and methods

### Cell culture and antibodies

The chemicals for buffer preparation, including SDS, Tris base, DMSO, and NaCl, were obtained from Sigma-Aldrich (St. Louis, MO). Cell culture medium and supplements, such as RPMI-1640/DMEM medium and Fetal Bovine Serum (FBS), were purchased from Invitrogen (Grand Island, NY). Human OSCC cells, including SCC9, SCC15, SCC25, and CAL27, were purchased from American Type Culture Collection (ATCC, Manassas, VA). CAL27 was established from tissue taken prior to treatment from a 56-year-old Caucasian male with a lesion of the middle of the tongue. CAL27 cells are epithelial, polygonal with highly granular cytoplasm. SCC25 cell was derived from a tongue squamous cell carcinoma obtained from a 70-year-old male patient. The immortalized epithelial and fibroblast cells, including HBE, NL20, MRC5, Het-1A, and FHC, were purchased from ATCC. The immortalized non-tumorous cell LO2 was obtained from the Cell Bank of the Chinese Academy of Sciences (Shanghai, China). The immortalized oral epithelial cell hTERT-OME was purchased from Applied Biological Materials (ABM) Inc. (Richmond, BC, Canada). The cells were maintained in a 37 °C humidified incubator with 5% CO_2_ following the standard protocols, and the mycoplasma test was performed every 2 months. The ionizing-radiation-acquired resistance cell lines CAL27-IR and SCC25-IR were established in our laboratory by exposing CAL27 and SCC25 cells to gradually increasing dose of ionizing radiation for approximately 6 months^13^. Briefly, CAL27 and SCC25 cells were serially irradiated with 2 Gy of X-rays to a final dose of 80 Gy. Cells were cultured with DMEM medium and maintained in a 37 °C humidified incubator with 5% CO_2_. The irradiated cells were passaged into new culture flask when growing to approximately 80% confluence. Reirradiation of X-rays was repeated over a period of 6 months. Antibodies against Aurora B (#3094, 1:1000), cleaved-caspase 3 (#9661, 1:1000), β-actin (#3700, 1:10,000), Histone H3 (#4499, 1:2000), p-Histone H3 Ser10 (#9701, 1:1000), and cleaved-PARP (#5625, 1:1000) were purchased from Cell Signaling Technology, Inc. (Beverly, MA). Antibodies against Ki67 (ab16667, 1:300) and p-Aurora B Thr232 (ab115793, 1:1000) were obtained from Abcam (Cambridge, UK). The ECL substrate (#32132, Thermo Fisher Scientific) was used for protein visualization.

### MTS assay

The MTS assay was performed as described previously^14^. Briefly, the cells were seeded (3 × 10^3^/well/100 mL) into a 96-well plate and treated with Tan IIA for various time points. The MTS reagent (Promega, Madison, WI) was added to the cell culture medium and incubated for 1 h according to the standard procedures.

### Soft agar assay

The soft agar assay was performed as described previously^15^. Briefly, OSCC cells were counted and suspended at a concentration of 8 × 10^3^ per well and seeded into a 6-well plate with 0.3% Basal Medium Eagle agar containing 10% FBS and Tan IIA. The cells were maintained in a 37 °C humidified incubator with 5% CO_2_ for 2 weeks. The colony was counted (diameter over 50 μm) with a light microscope.

### Clinical tissue sample collections

A total of 20 cases of OSCC tissues and matched adjacent non-tumor tissues were collected from 20 patients with written informed consent by the Department of pathology, The Third Xiangya Hospital of Central South University, Changsha, Hunan, China. All the patients received no treatment before surgery. The samples were frozen in liquid nitrogen, and protein was prepared for Western blotting analysis.

### Construction of Aurora B knockdown stable cell line

Construction of Aurora B knockdown stable cell line was performed as described previously^16^. The guaranteed sh-Aurora B lentivirus plasmids (Cat#1:V3SH11240-230132624, Cat#1:V3SH11240-225176452) were purchased from GE Horizon (Lafayette, CO). For the virus package, the sh-Aurora B lentivirus plasmid, psPAX2, and pMD2.G were co-transfected into 293T cells. The virus-containing supernatant was collected at 48 h after transfection. After centrifuge, the supernatant was filtered through a 0.45-μm filter and infected with OSCC cells with 8 µg/mL polybrene overnight. The fresh medium containing 1 μg/mL puromycin was added into the virus-infected OSCC cells and maintained for 1 week for stable cell selection.

### Molecular modeling

The Natural Product Library (Cat. no. L1400-01/02) is a product of Selleck Chemicals (Houston, TX), and the 74 compounds of interest (Table S1) used for screening were selected from this Natural Product Library. To find inhibitors against Aurora B, the X-ray crystal structure of Aurora B (PDB ID: 4C2V) was downloaded from Protein Data Bank1 (PDB). The protein structure was prepared using the Protein Preparation Wizard in Schrödinger Suite 2013, including filling in missing side chains, adding hydrogens, and minimizing heavy atoms with default parameters, before the corresponding protein grid files were generated for docking. Then the structure file of the nature product was pretreated in the LigPrep module of Schrödinger Suite 2013, and docking was performed based on the standard precision mode of Glide with default settings. Prime was employed to refine the binding pose further and calculate binding free energy by the MM-GBSA method in Schrödinger Suite 2013. Residues with distances from the ligand less than 12.0 Å were set as flexible. Other settings were kept in default. The docking pose for the receptor–ligand complex was then submitted to binding mode analysis and figure generation using PyMOL2. Herein, we could obtain the top-scored representative list. Barasertib was used as a positive control. To further confirm which top-scored representative exhibited a significant anti-tumor effect, the CAL27 cells were seeded in a 96-well plate and treated with a single dose of 2 μM natural compounds or DMSO (control) for 24 h. Cell viability was determined by MTS assay.

### Immunoblotting

Immunoblotting (IB) analysis was performed as described previously^17^. Briefly, cell lysate was prepared with RIPA buffer (#89900, Thermo Fisher Scientific) supplied with protease inhibitors and concentrated using the BCA protein assay kit (#23225, Thermo Fisher Scientific). Cell lysate (20 μg) was boiled with loading buffer at 95 °C for 5 min and subjected to SDS-PAGE electrophoresis. The protein was transferred onto polyvinylidene difluoride membranes and blocked with 5% non-fat milk at room temperature for 1 h, followed by subsequent incubation with primary antibody (overnight, 4 °C) and second antibody (1 h, room temperature). The target protein was visualized with the ECL chemiluminescence reagents (#32132, Thermo Fisher Scientific).

### Immunofluorescence

Immunofluorescence (IF) analysis was performed as described previously^18^. OSCC cells seeded in chamber slides, treated with Tan IIA, and fixed in 4% paraformaldehyde and permeabilized in 0.5% Triton X-100 for 30 min. The cells were blocked with 50% goat serum albumin in PBS and incubated with the primary antibody in a humidified chamber overnight at 4 °C, followed by incubation with the fluorescence-labeled second antibody at room temperature for 40 min. DAPI was used for counterstaining. The stained cells were viewed and captured with the confocal fluorescence microscope system (Nikon C1si; NIKON Instruments Co.).

### Ex vivo pull-down and in vitro ATP competitive binding assays

The ex vivo pull-down and in vitro ATP competitive binding assays were performed as described previously^19^. Briefly, Tan IIA was conjugated with Sepharose 4B beads (GE Healthcare Biosciences) following the standard procedure. The control Sepharose 4B beads or Tan IIA-conjugated Tan IIA-Sepharose 4B was incubated with CAL27 cell lysate (400 μg) overnight at 4 °C. The beads were raised with binding buffer and subjected to IB analysis. For in vitro ATP competitive binding assay, the active Aurora B kinase (Millipore, Burlington, MA) was incubated with various concentrations of ATP overnight at 4 °C, followed by the Sepharose 4B beads or Tan IIA-conjugated Sepharose 4B was added to the reaction and incubated for another 4 h at 4 °C. The beads were raised with wash buffer and subjected to IB analysis.

### In vitro Aurora B kinase assay

The in vitro Aurora B kinase assay was performed as described previously^20^. Briefly, 100 ng of active Aurora B kinase (Millipore) and 1 µg of the substrate (Histone H3 or Survivin, Millipore) were incubated with various doses of Tan IIA or Barasertib (positive control) in a 20 µL reaction containing 100 µM ATP and 1× kinase buffer (Cell Signaling Technology). The in vitro kinase assay was conducted at 30 °C for 30 min and stopped by boiling with the loading buffer at 95 °C for 5 min. Histone H3 phosphorylation was examined by IB analysis.

### Flow cytometry

The flow cytometry assay for cell cycle and apoptosis analysis was performed as described previously^21^. Briefly, the OSCC cells were treated with Tan IIA for 24 h and fixed with 70% ice-cold ethanol at 4 °C for 24 h. The cells were washed with PBS and stained with ribonuclease A-containing propidium iodide (50 μg/mL). The cell cycle was analyzed by flow cytometry. For apoptotic cell analysis, the OSCC cells were treated with Tan IIA for 72 h and suspended with 300 μL of binding buffer and incubated with 5 μL of Annexin V-FITC and propidium iodide-containing staining buffer at room temperature for 15 min at dark. The apoptotic cell was analyzed by FACS.

### Xenograft mouse model

The animal experiments were approved by the Institutional Animal Care and Use Committee of Central South University (Changsha, China). The OSCC xenograft models were constructed by s.c. injection of CAL27 (2 × 10^6^) or SCC25 (3 × 10^6^) cells into the right flank of 6-week-old athymic nude mice (*n* = 5). Tumor volume and mouse body weight were recorded every 2 days. The tumor-bearing mice were randomly assigned to parallel groups and initiated with Tan IIA treatment when the tumor volume reached around 100 mm^3^. The control group was administered vehicle control, whereas the compound-treated group was administered Tan IIA (low dose, 10 mg/kg; high dose, 30 mg/kg) every 2 days by i.p. injection. For irradiation treatment, the tumor-bearing mice were randomly divided into four groups (*n* = 6) when the tumor volume reached around 100 mm^3^: 1, vehicle control (0.5% dimethyl sulfoxide, 100 μL/every 2 days, i.p.); 2, local ionizing radiation (2 Gy/twice per week, irradiated with X-rays using X-RAD 320, Precision X-ray, Inc.,); 3, Tan IIA (30 mg/kg/every 2 days, i.p.); 4, Tan IIA (30 mg/kg/every 2 days, i.p.) + local ionizing radiation (2 Gy/twice per week). Tumor volume was determined according to the following formula: length × width × width/2. At the endpoint, tumor mass was fixed and subjected to immunohistochemistry (IHC) staining.

### Immunohistochemistry

IHC was performed as described previously^22^. Briefly, the xenograft tumor tissue was fixed with formalin and embedded in paraffin. For IHC staining, the tissue was deparaffinized by baking in a 60 °C incubator for 1 h and subsequently immersing into xylene to complete removal of paraffin. Tumor tissue was rehydrated by a graded series of ethanol, and Antigen retrieval was performed by immersing into the sodium citrate buffer (10 mM, pH 6.0) and boiled for 10 min. The 3% H_2_O_2_ in methanol was used for deactivating the endogenous horseradish peroxidase. Tumor tissue was washed with PBS, blocked with 50% goat serum albumin, and incubated with the primary antibody overnight at 4 °C in a humidified chamber. After incubation with the secondary antibody for 45 min and washed with PBS, the target protein was visualized with the DAB substrate and counterstained with hematoxylin.

### Blood analysis

Mouse blood was collected by cardiac puncture into the EDTA-coated tubes. The red blood cells (RBC), white blood cells (WBC), hemoglobin (Hb), alanine aminotransferase (ALT), aspartate aminotransferase (AST), and blood urea nitrogen (BUN) were analyzed at the Laboratory of the Third Xiangya Hospital of Central South University (Changsha, China).

### Statistical analysis

The SPSS 16.0 (SPSS, Inc, Chicago, IL) software was used for statistical analysis. All quantitative data were expressed as means ± SD as indicated. The significant differences between examined groups were determined by Student’s *t*-test or one-way ANOVA, and a probability value of less than 0.05 (*p* < 0.05) was used as the criterion for statistical significance.

## Results

### Aurora B is overexpressed in OSCC tumor tissue

**Fig. 1:**
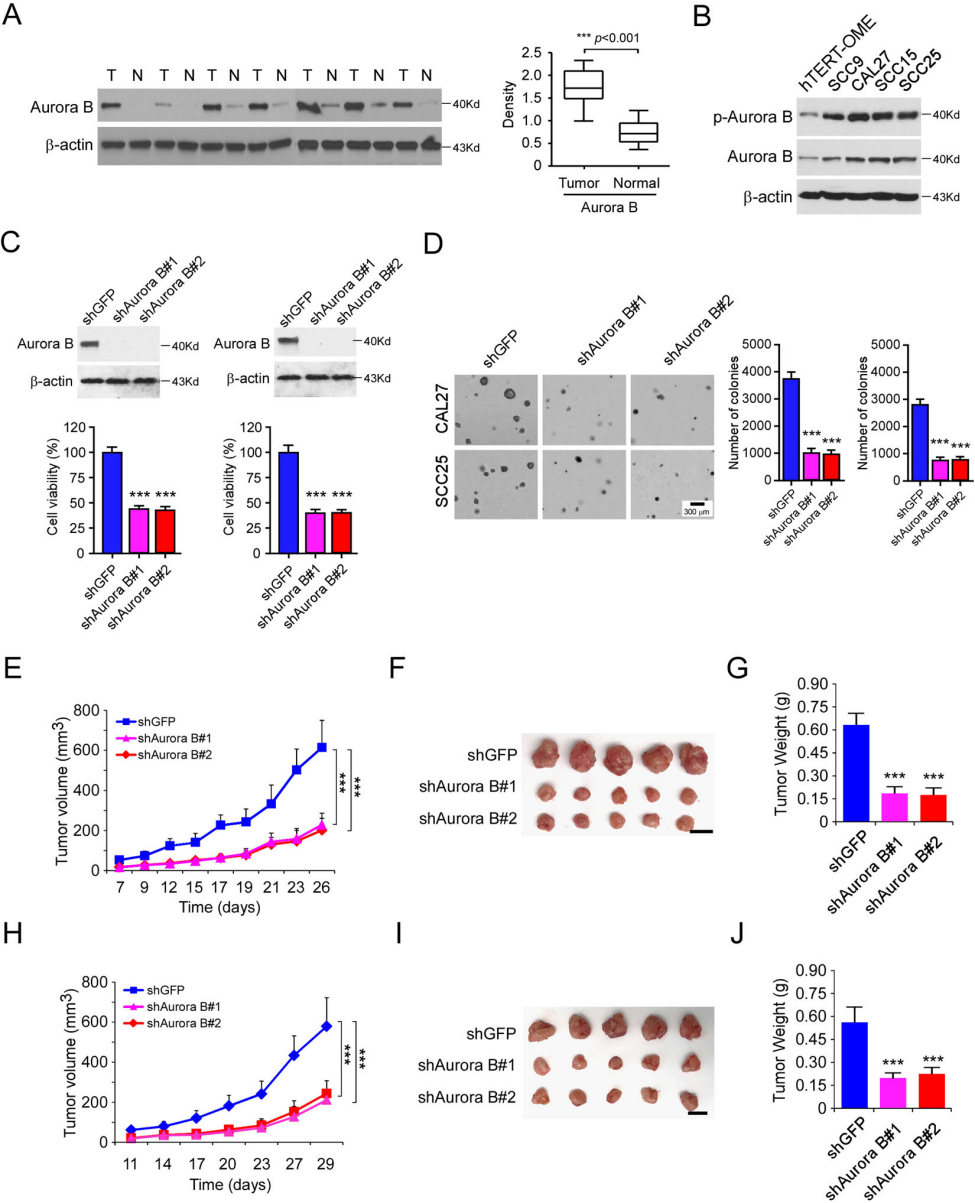
Aurora B is overexpressed in OSCC tumor tissue. **A** The representative immunoblotting (IB) results of Aurora B expression in 20 cases of matched OSCC patient tissues and adjacent non-tumor tissues (Mann–Whitney *U* test). T, tumor; N, adjacent non-tumor tissue. **B** IB analysis of Aurora B expression in hTERT-OME and OSCC cell lines. **C** Cell viability of SCC25 (left) and CAL27 (right) cells expressing shGFP or shAurora B. Top, IB analysis of Aurora B expression. Bottom, MTS analysis of cell viability. **D** Colony formation of CAL27 and SCC25 cells expressing shGFP or shAurora B; *n* = 3 independent biological replications, one-way ANOVA. Scale bar, 300 μm. **E**–**G** In vivo tumor growth of CAL27 cells expressing of shGFP or shAurora B. The tumor volume (**E**), the image of the tumor mass (**F**), and the weight of the tumor mass (**G**) of CAL27 xenograft tumors; *n* = 5 mice per group, one-way ANOVA. **H**–**J** In vivo tumor growth of SCC25 cells expressing of shGFP or shAurora B. The tumor volume (**H**), the image of the tumor mass (**I**), and the weight of the tumor mass (**J**) of SCC25 xenograft tumors; ***p < 0.001; *n* = 5 mice per group, one-way ANOVA. For **F** and **I**, scale bar, 1 cm.

We first examined the protein level of Aurora B in 20 cases of paired OSCC and adjacent tissues. Our data showed that Aurora B is highly expressed in OSCC tumor tissues when compared to that of the matched adjacent non-tumor tissues (Fig. 1A). Furthermore, Aurora B is overexpressed in all tested OSCC cell lines, including CAL27, SCC9, SCC15, and SCC25 (Fig. 1B). The IB data revealed that the shAurora B expressing CAL27 and SCC25 cells exhibited a robust reduction of Aurora B protein level (Fig. 1C). Moreover, knockdown of Aurora B caused a decrease of survivin (Supplementary Fig. 1A), reduction of cell viability (Fig. 1C), and colony formation (Fig. 1D). The in vivo data showed that knockdown of Aurora B significantly delayed the growth of CAL27-shAurora B- (Fig. 1E–G) and SCC25-shAurora B- (Fig. 1H–J) derived xenograft tumors. The shAurora B-expressing xenografts exhibited a dramatic decrease in tumor volume and tumor weight. Furthermore, knockdown of Aurora B significantly reduced the in vivo Edu incorporation (Supplementary Fig. 1B). The Western blot data indicated that the protein level of p-Aurora B and p-Histone H3 were attenuated in the shAurora B xenografts (Supplementary Fig. 1C). These data suggest that Aurora B is highly expressed in OSCC tissues and cell lines, and depletion of Aurora B delayed the tumor growth in vitro and in vivo.

### Tan IIA is a potential Aurora B kinase inhibitor

**Fig. 2:**
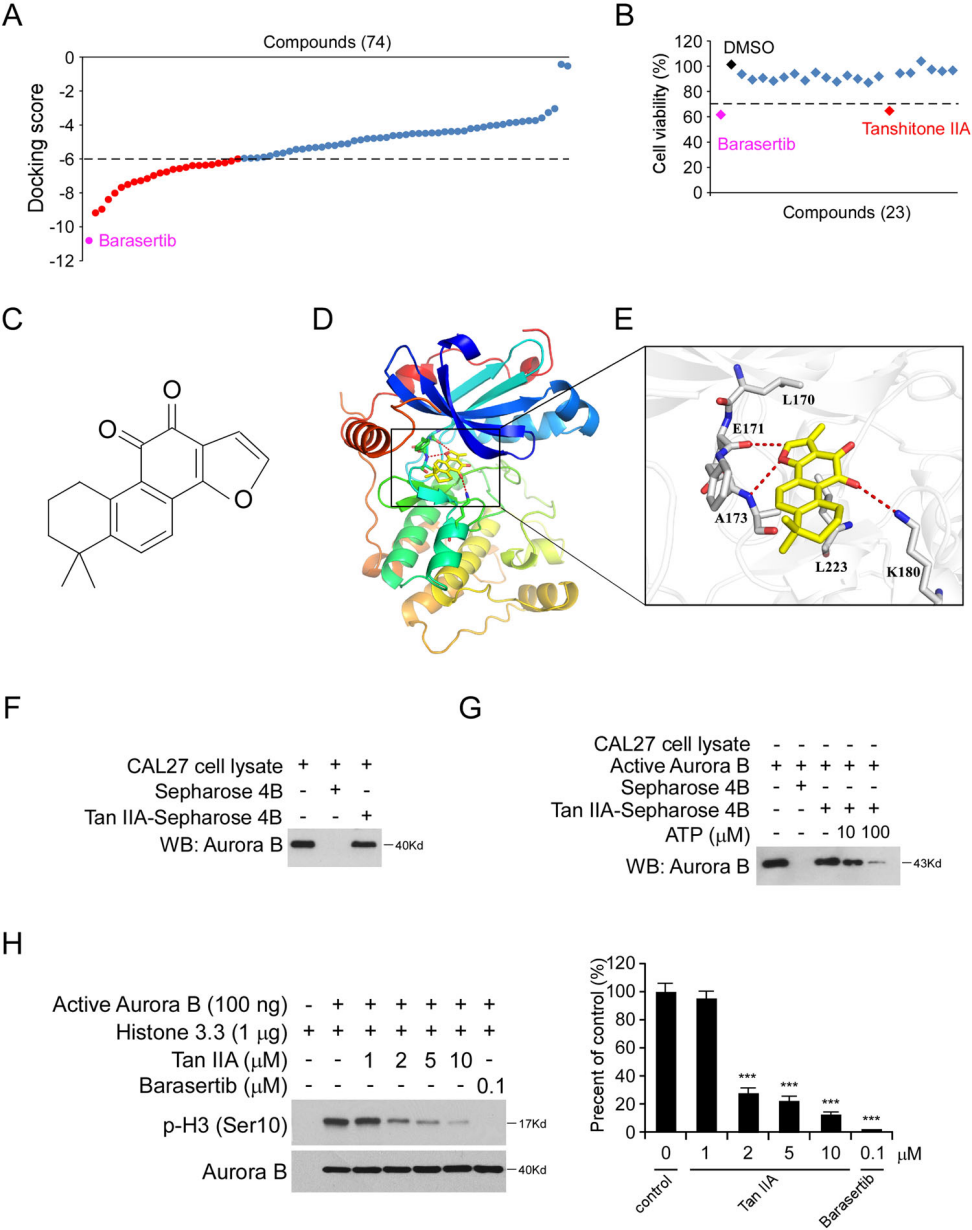
Tan IIA is an Aurora B kinase inhibitor. **A** The docking scores of the screened natural products. Barasertib was used as a positive control. Pink, Barasertib; red, the 23 nature products with a docking score less than −6. **B** MTS assay analysis of the cell viability of CAL27 cells with the 23 nature products treatment. **C** The chemical structure of Tanshinone IIA (Tan IIA). **D** The cartoon representation of the Tan IIA binding pocket in Aurora B. **E** Details of the binding mode of Tan IIA with Aurora B. The compound was shown as yellow sticks, while the proteins were depicted in cartoon representation with key residues labeled and demonstrated as gray sticks. Hydrogen bonds were shown as red dashed lines. **F** Tan IIA directly binds with Aurora B in CAL27 cells ex vivo. The ex vivo pull-down assay was performed as described in “Material and methods”, the beads were boiled and subjected to IB analysis. **G** The increase of ATP concentration inhibits the interaction between Tan IIA and Aurora B. **H** The in vitro kinase assay analyzes the effect of Tan IIA on Aurora B kinase activity. Barasertib was used as a positive control; *n* = 3 independent biological replications, one-way ANOVA; ***p < 0.001.

To discover some natural compounds that can inhibit OSCC cells through targeting Aurora B kinase activity, we performed a computational screening with 74 compounds of interest from the Selleck Chemicals (Houston, TX) natural products library (Supplementary Table 1). Our data showed that the commercial Aurora B kinase inhibitor, Barasertib, exhibited the most substantial inhibitor potential. Furthermore, as shown in Fig. 2A, we found that a total of 23 compounds showed a docking score of less than −6 (Fig. 2A and Supplementary Table 1). To determine which top-scored representative exhibited the most potent anti-tumor effect in OSCC cells, we performed the MTS assay to examine the cell viability of CAL27 cells with these 23 compounds treatment. The result showed that only Tan IIA and Barasertib reduced cell viability by over 30% compared to the DMSO control (Fig. 2B). We therefore focus on Tan IIA (Fig. 2C) for further study.

The molecular modeling result showed that Tan IIA was docked into the ATP-binding pocket of Aurora B. We then further refined the docking poses for binding free energy (Δ*G*) calculation. Tan IIA showed a relatively favorable docking score of −6.806 and Δ*G* of −49.979, indicating that Tan IIA could inhibit Aurora B by competitively binding at the ATP-binding site (Fig. 2D). As shown in Fig. 2E, the furan oxygen of this compound was predicted to form hydrogen bonding interaction with the backbone nitrogen of A173 in the hinge region. Meanwhile, an aromatic hydrogen bond was demonstrated between E171 and this furan substructure. In the solvent accessible region, the positively charged amino of K180 also tended to form hydrogen bonding with the electro-withdraw carbonyl group of Tan IIA, which further strengthened the affinity. In addition, Tan IIA adopted broad hydrophobic interactions with other residues in the pocket, such as L170 and L223. These results suggested that Tan IIA could be an ATP-competitive inhibitor of Aurora B. To validate this molecular modeling data, we next determined the interaction between Aurora B and Tan IIA. Our data showed that the Tan IIA-conjugated Sepharose 4B beads, but not the Sepharose 4B beads only, can bind with Aurora B in vitro (Fig. 2F). Moreover, with the increase of ATP concentration, the interaction between Tan IIA and Aurora B was decreased, indicating there is a competition binding between Tan IIA and ATP with Aurora B protein (Fig. 2G). In addition, the in vitro Aurora B kinase assay showed that Tan IIA inhibited Aurora B kinase activity in a dose-dependent manner, as the phosphorylation of histone H3 on Ser10 and survivin on Thr117 were significantly decreased with the graded increase of Tan IIA concentration (Fig. 2H and Supplementary Fig. 1D). Furthermore, although the Aurora C kinase has redundant functions and substrates as Aurora B, the in vitro kinase assay showed that Tan IIA could not inhibit Aurora C activity significantly (Supplementary Fig. 1E). All of these data indicate that Tan IIA is an Aurora B kinase inhibitor.

### Tan IIA suppresses OSCC cells and exhibits little cytotoxicity

**Fig. 3:**
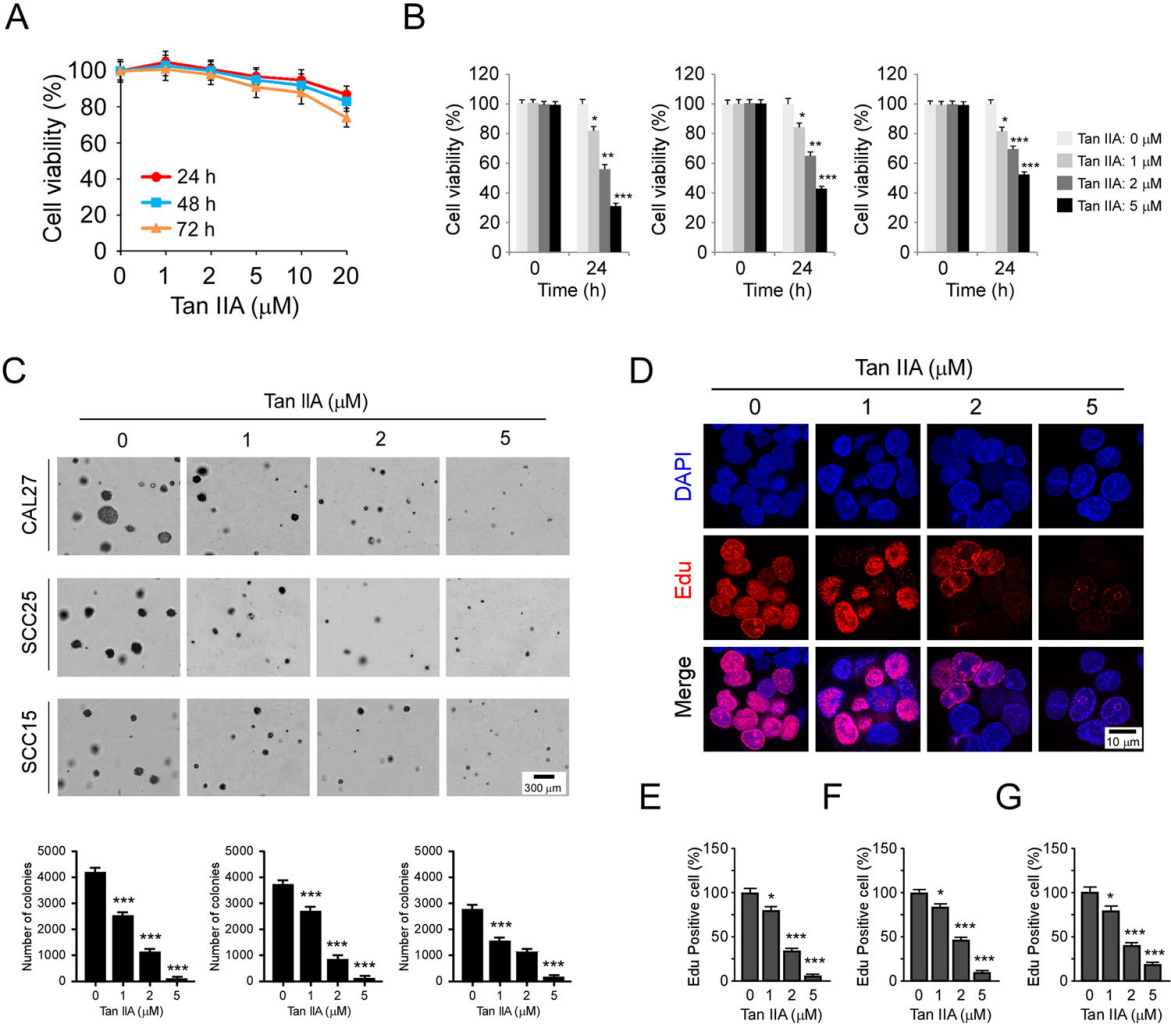
Tan IIA suppresses OSCC cells. **A** MTS assay analyzes the cell viability of hTERT-OME cells with Tan IIA treatment for various time points. **B** MTS assay analyzes the cell viability of CAL27 (left), SCC25 (middle), and SCC15 (right) cells with Tan IIA treatment for 24 h; *n* = 3 independent biological replications, one-way ANOVA. **C** Soft agar assay examines the colony formation of CAL27 (top), SCC25 (middle), and SCC15 (bottom) cells treated with Tan IIA; *n* = 3 independent biological replications, one-way ANOVA; *p < 0.05, **p < 0.01, ***p < 0.001. **D**–**G** Edu incorporation assay and quantitative analysis of the effect of Tan IIA on OSCC proliferation. (**D**) The representative image of Edu incorporation assay for CAL27 cells. (**E**–**G**) Quantitative analysis of Edu incorporated cells for CAL27 (**E**), SCC25 (**F**), and SCC15 (**G**); *n* = 3 independent biological replications, one-way ANOVA; *p < 0.05, **p < 0.01, ***p < 0.001.

We next determined whether Tan IIA exerted any cytotoxic effect on immortalized oral epithelial cells. The hTERT-OME cells were treated with various doses of Tan IIA for 24, 48, and 72 h, cell viability was examined by MTS assay. The result showed that Tan IIA had no significant cytotoxicity against hTERT-OME cells while concentrations were <20 μM (Fig. 3A). Moreover, the MTS data revealed that Tan IIA did not reduce the cell viability of a panel of immortalized non-tumor cells, including immortalized lung epithelial cells HBE and NL20, immortalized lung fibroblast cell MRC5, immortalized hepatocytes LO2, colon epithelial cell FHC, and esophageal epithelial cell Het-1A (Supplementary Fig. 2). To further confirm the anti-tumor effect of Tan IIA on OSCC, we examined the inhibitory effect of Tan IIA on various OSCC cell lines, including CAL27, SCC25, and SCC15. The results revealed that Tan IIA reduced the cell viability of OSCC cells in a dose-dependent manner, and Tan IIA inhibited CAL27 cells most significantly (Fig. 3B). The results of colony formation assay indicated that CAL27 cells formed the largest size of colony in soft agar. Treatment with Tan IIA reduced both colony number and colony size, and 5 μM Tan IIA almost blocked the colony formation of these tested OSCC cells (Fig. 3C). Edu incorporation assay showed that Tan IIA reduced the efficacy of Edu incorporation in CAL27, SCC25, and SCC15 cells (Fig. 3D–G). These results indicate that Tan IIA inhibits OSCC cells dose-dependently but exhibits no significant toxicity against immortalized oral epithelial cells.

### Tan IIA inhibits Aurora B activity and induces G2/M phase cell cycle arrest in OSCC cells

**Fig. 4:**
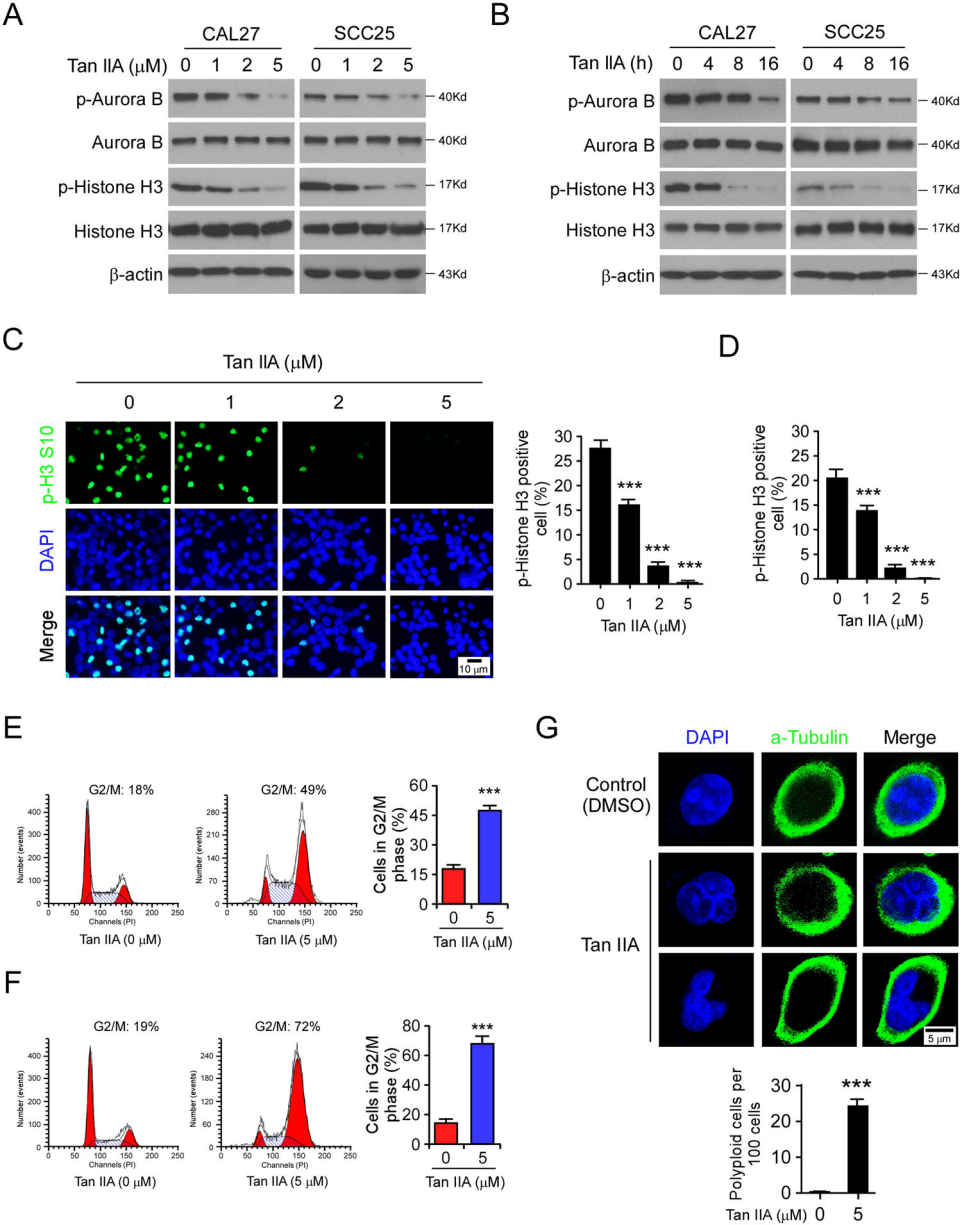
Tan IIA inhibits Aurora B activity and induces G2/M phase cell cycle arrest in OSCC cells. **A** IB analysis of p-Aurora B and p-Histone H3 Ser10 in Tan IIA-treated CAL27 and SCC25 cells with various doses for 24 h. **B** IB analysis of p-Aurora B and p-Histone H3 Ser10 in 2 μM Tan IIA-treated CAL27 and SCC25 cells for different time points. **C** and **D** Immunofluorescence (IF) analysis of the phosphorylation of Histone H3 Ser10 in Tan IIA-treated CAL27 (**C**) and SCC25 cells (**D**); *n* = 3 independent biological replications, one-way ANOVA. ***p < 0.001. Scale bar, 10 μm. **E** and **F** Cell cycle analysis by flow cytometry of Tan IIA-treated CAL27 (**E**) and SCC25 cells (**F**); *n* = 3 independent biological replications, Student’s *t* test. ***p < 0.001. **G** IF analysis of the population of polyploidy cells in CAL27 cells treated with Tan IIA for 48 h. Scale bar, 5 μm; *n* = 3 independent biological replications, Student’s *t* test, ***p < 0.001.

To confirm the inhibitory effect of Tan IIA on Aurora B kinase activity in OSCC cells, we analyzed the phosphorylation of histone H3 (Ser10) with Tan IIA treatment. The results showed that Tan IIA reduced the phosphorylation of Aurora B kinase on Thr232 and histone H3 on Ser10 dose-dependently (Fig. 4A). Moreover, the time-course experiments revealed that Tan IIA decreased the phosphorylation of both Aurora B and histone H3 in a time-dependent manner (Fig. 4B). Likewise, the IF staining showed that Tan IIA significantly reduced the population of p-Histone H3 (Ser10) positive cells in CAL27 (Fig. 4C) and SCC25 cells (Fig. 4D). Consistently, with the inhibition of Aurora B kinase activity, treatment with Tan IIA induced a significant cell cycle arrest at the G2/M phase in OSCC cells, and 5 μM Tan IIA caused nearly 50% of OSCC cells G2/M arrest (Fig. 4E, F). Inhibition of Aurora B kinase results in cytokinesis failure and abnormal exit from mitosis, which eventually leads to polyploidy cells. Our data revealed that treatment with 5 μM Tan IIA caused the induction of polyploidy cells in CAL27 cells, whereas no polyploidy cells were observed in the DMSO control (Fig. 4G). Also, Tan IIA significantly increased the population of the mitotic cells with an abnormal multipolar spindle or misaligned chromosomes (Supplementary Fig. 3). These results indicate that Tan IIA inhibits Aurora B activity and induces G2/M phase cell cycle arrest and polyploidy cells formation in OSCC cells.

### Tan IIA promotes apoptosis in OSCC cells

**Fig. 5:**
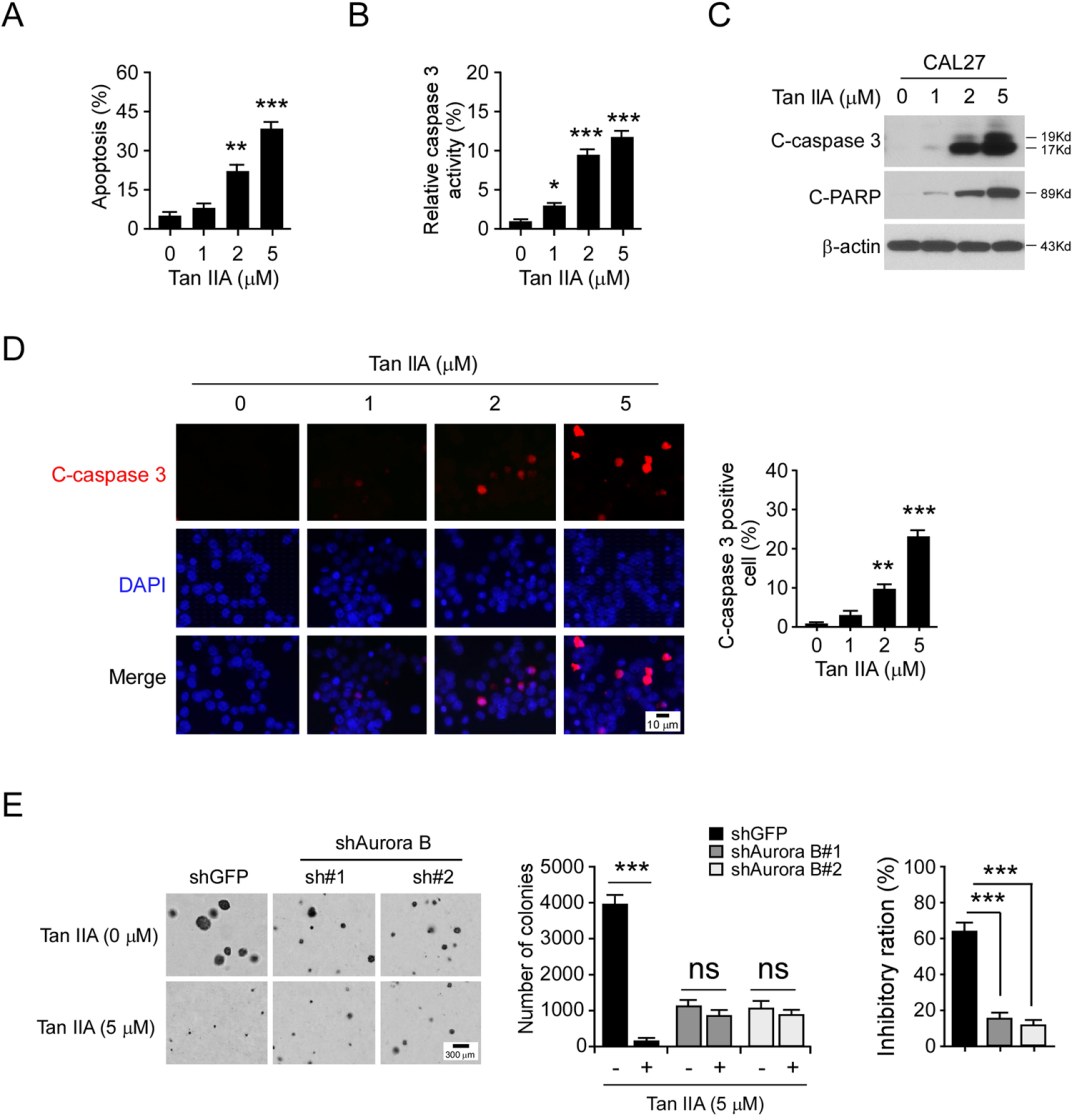
Tan IIA promotes apoptosis in OSCC cells. **A** Flow cytometry examination of apoptotic CAL27 cells with Tan IIA treatment for 72 h; *n* = 3 independent biological replications, one-way ANOVA; **p < 0.01, ***p < 0.001. **B** and **C** CAL27 cells were treated with Tan IIA for 72 h, cell lysate was subjected to cleaved-caspase 3 activity analysis (**B**) and IB analysis (**C**). For **B**, *n* = 3 independent biological replications, one-way ANOVA; *p < 0.05, ***p < 0.001. **D** CAL27 cells were treated with Tan IIA for 72 h and subjected to IF analysis with cleaved-caspase 3 antibody. Scale bar, 10 μm; *n* = 3 independent biological replications, one-way ANOVA; **p < 0.01, ***p < 0.001. **E** Knockdown of Aurora B decreased the sensitivity to Tan IIA. Soft agar assay analysis of the anti-tumor effect of Tan IIA on shGFP and shAurora B-expressing CAL27 cells (left and middle), the inhibitory ratio of Tan IIA on colony formation was calculated (right); *n* = 3 independent biological replications, two-way ANOVA, ***p < 0.001; ns, not statistically significant.

Because Tan IIA caused the induction of polyploidy cells, we therefore determined whether Tan IIA promotes apoptosis. The flow cytometry data showed that treatment with Tan IIA for 72 h increased the population of apoptotic CAL27 cells dose-dependently (Fig. 5A). Furthermore, the activity of cleaved-caspase 3 was enhanced with Tan IIA treatment (Fig. 5B). The result showed that the expression of cleaved-caspase 3 and -PARP were dramatically upregulated with Tan IIA treatment, which further confirmed the induction of apoptosis in CAL27 cells (Fig. 5C). We next performed the IF staining with cleaved-caspase 3 antibody. Consistently, the result revealed that the positive staining of cleaved-caspase 3 was significantly increased with Tan IIA treatment (Fig. 5D). These results indicate that Tan IIA caused apoptosis in OSCC cells. Furthermore, knockdown of Aurora B with shRNA significantly reduced the colony number and colony size in CAL27 cells. Moreover, the inhibitory ratio of Tan IIA on shGFP-expressing stable cells was over 60%, whereas this inhibitory efficacy was reduced to <20% in shAurora B expressing CAL27 cells (Fig. 5E). These results indicate that as a target protein, Aurora B plays a crucial role in the sensitivity of OSCC cells to the anti-tumor effect of Tan IIA.

### Tan IIA inhibits the in vivo tumor growth of OSCC cells

**Fig. 6:**
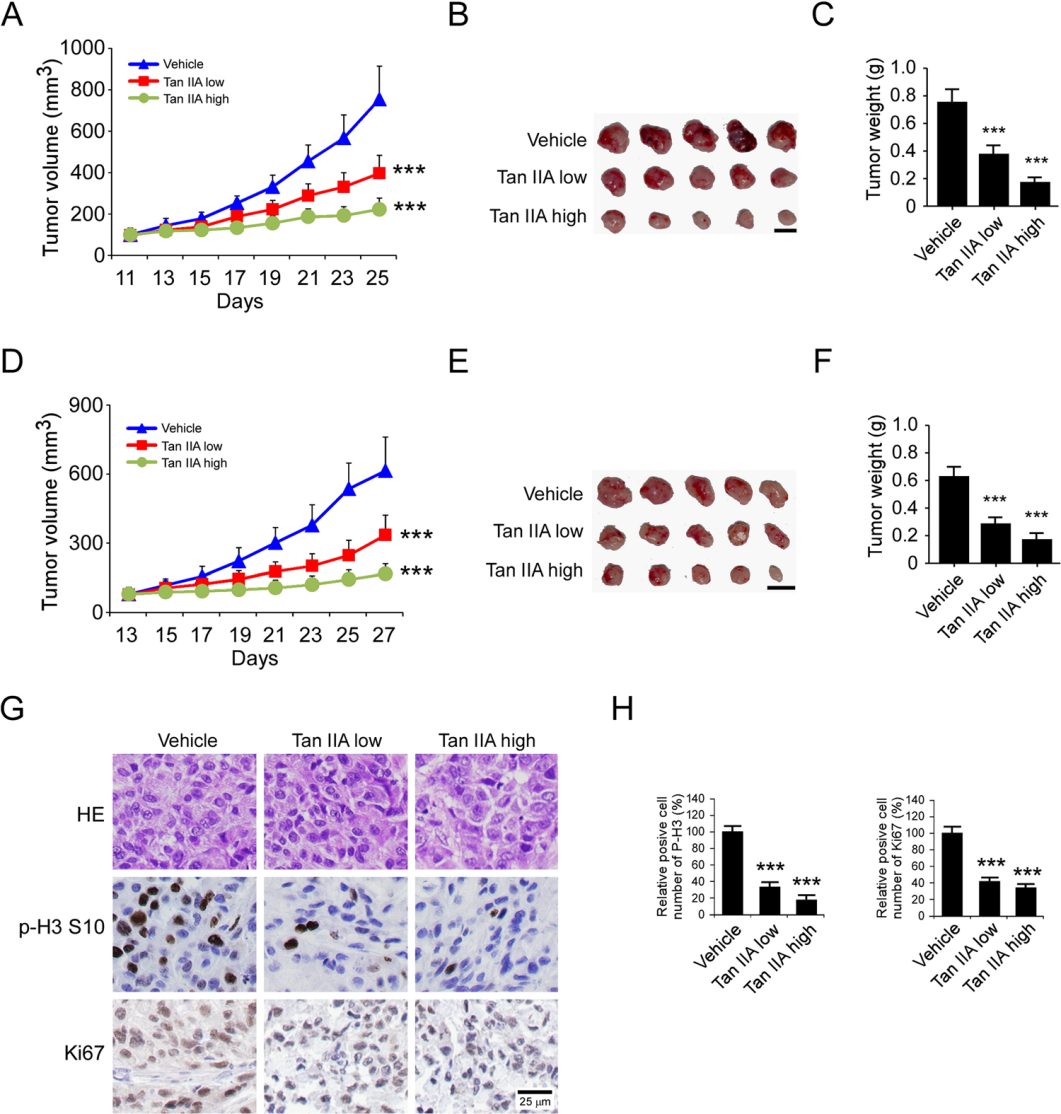
Tan IIA suppresses the in vivo tumor growth of OSCC cells. **A**–**C** The tumor volume (**A**), the image of tumor mass (**B**), and tumor weight (**C**) of CAL27-derived xenograft tumors treated with vehicle or Tan IIA; *n* = 5 mice per group, one-way ANOVA. ***p < 0.001. **D**–**F** The tumor volume (**D**), The image of tumor mass (**E**), and tumor weight (**F**) of SCC25-derived xenograft tumors treated with vehicle or Tan IIA; *n* = 5 mice per group, one-way ANOVA.***p < 0.001. For **B** and **E**, scale bar, 1 cm. **G** IHC staining of Ki67 and p-Histone H3 Ser10 in CAL27-derived xenograft tumors with vehicle or Tan IIA treatment. Scale bar, 25 μm. **H** Qualification analysis of p-Histone H3 Ser10 and Ki67 in CAL27-derived xenograft tumors with vehicle or Tan IIA treatment; one-way ANOVA, ***p < 0.001.

To further confirm the in vivo anti-tumor effect of Tan IIA, we performed the xenograft mouse model using CAL27 and SCC25 cells. The results showed that treatment with Tan IIA significantly reduced the tumor growth of CAL27, as the tumor volume in the vehicle- and Tan IIA-treated group was 756 ± 159 mm^3^, 397 ± 87 mm^3^ (low dose), and 223 ± 54 mm^3^ (high dose), respectively (Fig. 6A). Furthermore, the tumor weights were reduced by ~50% (low dose) and ~70% (high dose) in the Tan IIA-treated group (Fig. 6B, C). Consistently, a similar inhibitory effect was observed in Tan IIA-treated SCC25 xenograft tumors (Fig. 6D–F). The IHC staining results showed that Tan IIA reduced the population of Ki-67-positive cells, and decreased the protein level of Histone H3 Ser10 dose-dependently (Fig. 6G, H). Our data showed that Tan IIA did not cause a significant bodyweight loss of tumor-bearing mice at a dose of 30 mg/kg (Supplementary Fig. 4A). In addition, the blood analysis showed that the RBC and WBC count, as well as the Hb, AST, ALT, and BUN, were unaffected in Tan IIA-treated mice (Supplementary Fig. 4B). Histopathology of normal tissues from vital organs (heart, kidney, lung, spleen, liver) revealed no evidence of normal tissue toxicities after treatment with Tan IIA (Supplementary Fig. 4C). These data indicate that Tan IIA is well-tolerated in mice.

### Tan IIA overcomes radioresistance in OSCC cells

**Fig. 7:**
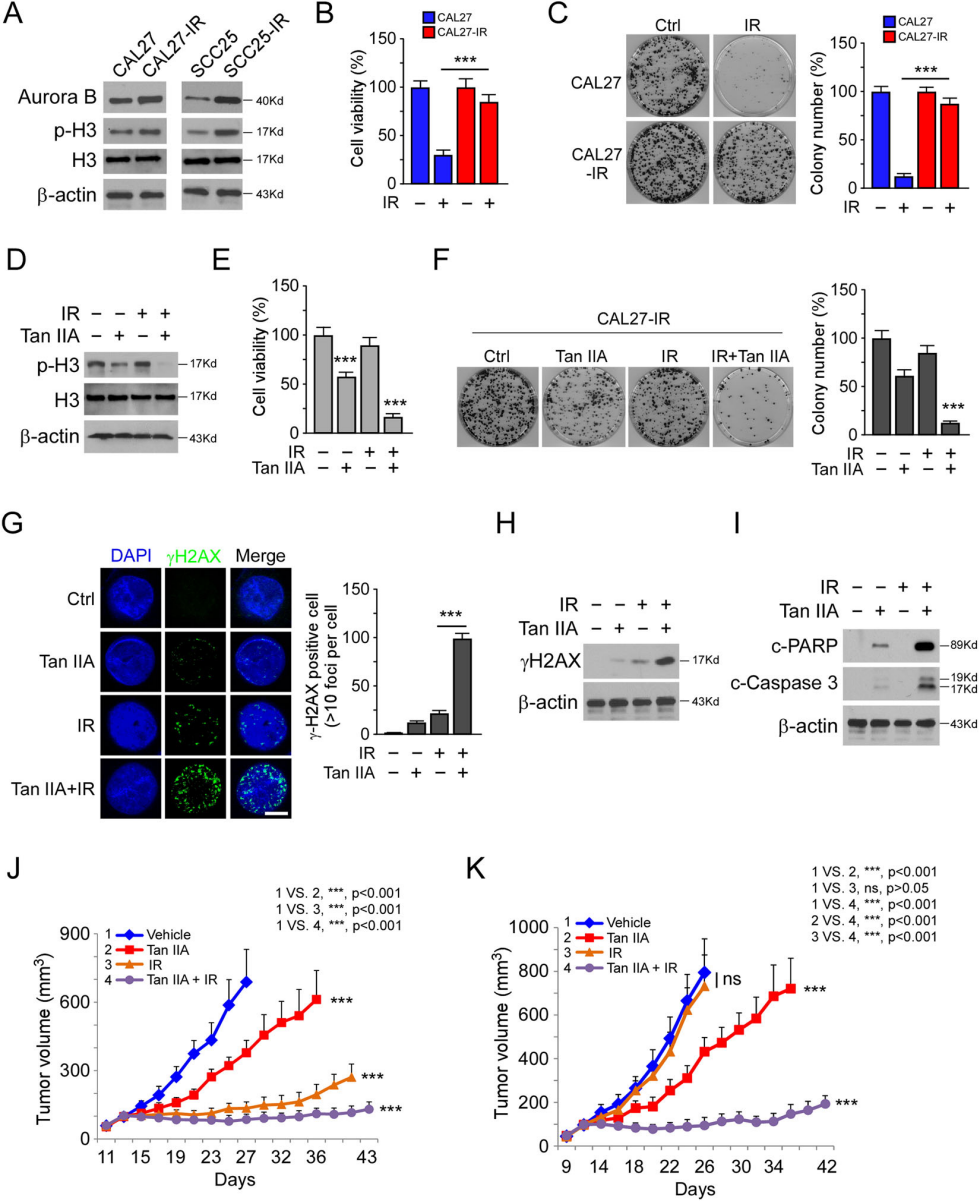
Tan IIA overcomes radioresistance in OSCC cells. **A** IB analysis of the protein level of Aurora B and p-H3 in CAL27/CAL27-IR and SCC25/SCC25-IR cells. **B** The effect of irradiation (IR) on cell viability of CAL27/CAL27-IR cells. CAL27 and CAL27-IR cells were treated with 4 Gy IR, cell viability was examined 72 h later by MTS assay; ***p < 0.001. **C** The effect of IR on colony formation of CAL27/CAL27-IR cells. CAL27 and CAL27-IR cells were treated with 4 Gy IR, colony number was examined 2 weeks later; ***p < 0.001. **D** IB analysis of the protein level of Aurora B and p-H3 in CAL27-IR cells treated with Tan IIA (2 μM), IR (4 Gy), or a Tan IIA/IR combination. **E** and **F** The cell viability (**E**) and colony formation (**F**) of CAL27-IR cells treated with Tan IIA (2 μM), IR (4 Gy), or a Tan IIA/IR combination; ***p < 0.001. **G** and **H** Immunofluorescence (**G**) or IB (**H**) analysis of γH2AX in CAL27-IR cells treated with Tan IIA (2 μM), IR (4 Gy), or a Tan IIA/IR combination. Scale bar, 5 μm; ***p < 0.001. **I** IB analysis of apoptosis in CAL27-IR cells treated with Tan IIA (2 μM), IR (4 Gy), or a Tan IIA/IR combination. **J** In vivo tumorigenesis of CAL27 cells treated with vehicle control, Tan IIA, IR, or a Tan IIA/IR combination; ***p < 0.001. **K** In vivo tumorigenesis of CAL27-IR cells treated with vehicle control, Tan IIA, IR, or a Tan IIA/IR combination; ***p < 0.001; ns, not statistically significant.

We next determine whether deregulation of Aurora B is related to radioresistance in OSCC cells. We examined the protein level of Aurora B in two pairs of radioresistant/parental OSCC cells, CAL27/CAL27-IR, and SCC25/SCC25-IR. The IB data showed that Aurora B is upregulated in radioresistant CAL27-IR and SCC25-IR cells when compared to the parental cells (Fig. 7A). Irradiation (4 Gy) significantly decreased the cell viability and colony formation of CAL27 and SCC25, but not that of the radioresistant CAL27-IR and SCC25-IR cells (Fig. 7B, C and Supplementary Fig. 5A, B). Treatment with Tan IIA or a combination of Tan IIA with irradiation reduced the phosphorylation of Histone H3 (S10) in CAL27-IR and SCC25-IR cells robustly (Fig. 7D and Supplementary Fig. 5C). In contrast, irradiation only did not cause a significant decrease of p-H3 in radioresistant OSCC cells. Consistently, Tan IIA, but not a single dose of irradiation, decreased the cell viability and colony formation of CAL27-IR and SCC25-IR cells (Fig. 7E, F and Supplementary Fig. 5D, E). Furthermore, pretreated with Tan IIA enhanced the anti-tumor efficacy of irradiation in the radioresistant cells (Fig. 7E, F and Supplementary Fig. 5D, E). The IF and IB results showed that Tan IIA enhanced irradiation-induced DNA damage, as the population of γ-H2AX-positive cells was increased substantially (Fig. 7G, H). In addition, the combination of Tan IIA with irradiation promoted apoptosis in CAL27-IR cells (Fig. 7I). We next determined whether Tan IIA overcomes radioresistance in vivo. CAL27/CAL27-IR xenograft tumors were treated with Tan IIA, irradiation, or in combination for 2 weeks. Our data revealed that CAL27-derived tumors were sensitive to irradiation with significantly delayed tumor development. In contrast, the CAL27-IR-derived xenografts were resistant to radiotherapy (Fig. 7J, K). Tan IIA inhibited the growth of both CAL27 and CAL27-IR-derived xenografts, and the combination of Tan IIA sensitized CAL27-IR xenograft to radiotherapy (Fig. 7J, K). A similar inhibitory effect was observed in SCC25 and SCC25-IR xenografts, and the combination of Tan IIA with radiotherapy significantly suppressed the in vivo tumor growth (Supplementary Fig. 5F, G). These results indicate that Tan IIA suppresses tumor growth and overcomes radioresistance.

## Discussion

As a critical kinase responsible for cell cycle progression and G2/M transition, Aurora B is frequently overexpressed in human cancer cells and closely related to poor prognosis^9^. In small cell lung cancer (SCLC), Aurora B is required for the survival of RB1-deficient cancer cells^23^. Suppression of Aurora B kinase promotes apoptosis in colorectal cancer cells^24^. The protein level of Aurora B is significantly overexpressed in prostate cancer^25^, gastric cancer^26^, and esophageal carcinoma^20^. Aurora B interacts with CHK2 and maintains mitotic activity to facilitate hepatocellular carcinoma progression^27^. Moreover, suppression of Aurora B enhances the efficacy of radiotherapy^28,29^ and overcomes acquired resistance in EGFR targeting therapy^30^. In addition, overexpression of Aurora B causes relapse of B cell acute lymphoblastic leukemia and blunts the glucocorticoid sensitivity^31^. Here, we found that Aurora B is highly expressed in human OSCC tumor tissues and cancer cells. Depletion of Aurora B by shRNA significantly decreased cell viability, colony formation, and in vivo tumor growth. In combination with radiation, Tan IIA overcomes radioresistance in OSCC xenograft tumors. These evidences suggest that Aurora B is a promising target for anti-cancer treatment.

The biological activities of Danshen have been well studied over the past few decades. As one of the most abundant lipophilic components isolated from Danshen, Tan IIA exhibits potential pharmacological activities on various human diseases, such as atherosclerosis and left ventricular hypertrophy. Recently, Tan IIA has gained increasing attention owing to the significant in vitro and in vivo anti-tumor efficacy in multiple tumor models^32,33^. Tan IIA suppresses both solid tumors and hematological malignancies, including non-small cell lung cancer^34^, prostate cancer^35^, colorectal cancer^36^, hepatocellular carcinoma^37^, and leukemia^38^. Treatment with Tan IIA induced cell cycle arrest, impaired angiogenesis, invasion, and metastasis, and promoted apoptosis^33^. Furthermore, Tan IIA enhanced the tumor-killing effect when combined with chemotherapy agents or radiotherapy^39,40^. Tan IIA sensitized the anti-tumor effects of irradiation on laryngeal cancer via the JNK pathway^41^. The combination of Tan IIA and doxorubicin possesses synergism and attenuation effects on doxorubicin in the treatment of breast cancer^42^. Tan IIA enhances chemosensitivity of 5-Fu in colon cancer cells by suppressing nuclear factor-κB^43^ and overcomes oxaliplatin resistance via inhibiting ERK/Akt signaling^36^. These results indicate that Tan IIA regulates DNA damage response. However, the underlying mechanisms need further elucidation. A panel of protein kinases has been demonstrated as potential targets in Tan IIA-treated tumor cells, such as PI3K/Akt, MAPK, and mTOR^40,44,45^. In the present study, with a molecular modeling screening, we identified that Tan IIA is a candidate inhibitor for Aurora B kinase in an ATP competitive binding-dependent manner. We validated this inhibitory effect in vitro, ex vivo, and in vivo and confirmed that Tan IIA inhibited the phosphorylation of Aurora B and downstream target Histone H3. Tan IIA induced G2/M cell cycle arrest, increased the population of polyploid cells, and eventually resulted in cell death in OSCC cells. Our results extend the anti-tumor mechanism of Tan IIA and indicate that Aurora B is an attractive target for OSCC treatment. Aurora A kinase contributes to the progression of OSCC through modulating epithelial-to-mesenchymal transition (EMT) and apoptosis^46^. We could not exclude the possibility that Tan IIA-induced tumor suppression may be caused by the Aurora A signaling inhibition indirectly.

Due to the crucial role in tumorigenesis, Aurora B has become a fantastic target for anti-cancer drug development. So far, a variety of Aurora B and pan Aurora kinase inhibitors have entered the clinical trials for solid tumors and refractory leukemia or myelofibrosis and exhibited significant tumor-killing efficacy^8^. The best-studied AuroraB kinase inhibitor Barasertib, also known as AZD1152, showed promising anti-tumor activity in AML. However, like other Aurora B kinase inhibitor, Barasertib has a narrow therapeutic index limited by toxicity, such as neutropenia^8,47^. Thus, further discovery of highly selective and effective Aurora B kinase inhibitors with fewer side effects are required for clinical treatment. Our data revealed that the natural product Tan IIA inhibited Aurora B kinase but had no apparent adverse effect in Tan IIA-treated mice, indicating that Tan IIA is well-tolerated in vivo. Importantly, Tan IIA is a major component of traditional herb Danshen and currently used for clinical treatment of arecoline- and areca nut extract-induced oral submucous fibrosis (OSF), an oral precancerous lesion. In addition, previous reports have shown that the mechanism underlying such a role of Tan IIA is related to the suppression of epithelial-mesenchymal transition and reactivation of p53 signaling^48,49^. Thus, Tan IIA is a candidate that hits targeting Aurora B kinase and deserves further study.

Overall, we demonstrated that Aurora B kinase is required for maintaining the tumorigenic properties of OSCC cells. Inhibition of Aurora B activity caused cell cycle G2/M arrest, polyploid cells, and ultimately apoptosis. The in vitro and in vivo anti-tumor effect indicates that Tan IIA has the potentials for OSCC treatment.

## Supplementary information

Supplementary figure legends

Supplementary table

Supplementary figure 1

Supplementary figure 2

Supplementary figure 3

Supplementary figure 4

Supplementary figure 5
