# Supplementary material for: Targeting Aurora B kinase with Tanshinone IIA suppresses tumor growth and overcomes radioresistance
Source: Cell Death Dis. 2021 Feb 4;12(2):152. doi: 10.1038/s41419-021-03434-z (PMC7862432; doi:10.1038/s41419-021-03434-z)
Supplement: Supplementary file 1 — Supplementary figure legends [file 41419_2021_3434_MOESM1_ESM.docx]

**Supplementary Figure legends**

**Supplementary Figure 1**. The effect of Aurora B knockdown in OSCC cells. A, Knockdown of Aurora B in CAL27 and SCC25 cells affects the expression of survivin. B, Edu incorporation assay determination of tumor cell proliferation in shGFP and shAurora B xenografts. n = 3 independent biological replications, one-way ANOVA. *** *p*<0.001. Scale bar, 25 μm. C, Immunoblotting (IB) analysis of Aurora B and Histone H3 phosphorylation in shGFP and shAurora B xenografts. D, The *in vitro* kinase assay analyzes the effect of Tan IIA on Aurora B kinase-mediated survivin phosphorylation. Barasertib serves as a positive control. one-way ANOVA. *** *p*<0.001. E, The *in vitro* kinase assay analyzes the effect of Tan IIA on Aurora C kinase-mediated Histone H3 phosphorylation. Danusertib serves as a positive control. one-way ANOVA. *** *p*<0.001.

**Supplementary Figure 2**. The effect of Tan IIA on immortalized non-tumor cells. A, MTS analyzes the cell viability of a panel of immortalized non-tumor cells treated with various doses of Tan IIA. ns, not statistically significant. B, IB analysis of the inhibitory effect of Tan IIA on Aurora B and Histone H3 phosphorylation.

**Supplementary Figure 3**. Treatment with Tan IIA causes the defections in mitosis. CAL27 cells were treated with Tan IIA for 24 h. Cells were fixed for immunofluorescence (IF) analysis with α-tubulin (left). Quantification analysis of mitotic cells with a bipolar spindle, multipolar spindle, or misaligned chromosomes (right). n = 3 independent biological replications, one-way ANOVA. *** *p*<0.001. Scale bar, 5 μm.

**Supplementary Figure 4**. Toxicity analysis of Tan IIA treated mouse. A, Body weights of CAL27 xenograft tumor-bearing mice with vehicle and Tan IIA treatment (low, 10 mg/kg; high, 30 mg/kg). B, Blood analysis of tumor-bearing mice with vehicle and Tan IIA treatment. C, H&E histology of various organs after various treatments for 25 days. Scale bar, 25 μm.

**Supplementary Figure 5**. Tan IIA overcomes radioresistance in OSCC cells. A, The effect of irradiation (IR) on cell viability of SCC25/SCC25-IR cells. SCC25 and SCC25-IR cells were treated with 4 Gy IR, cell viability was examined 72 h later by MTS assay. ****p*<0.001. B, The effect of IR on colony formation of SCC25/SCC25-IR cells. SCC25 and SCC25-IR cells were treated with 4 Gy IR, colony number was examined 2 weeks later. ****p*<0.001. C, IB analysis of the protein level of p-H3 in SCC25-IR cells treated with Tan IIA (2 μM), IR (4 Gy), or a Tan IIA/IR combination. D and E, The cell viability (D) and colony formation (E) of SCC25-IR cells treated with Tan IIA (2 μM), IR (4 Gy), or a Tan IIA/IR combination. ****p*<0.001. F, *In vivo* tumorigenesis of SCC25 cells treated with vehicle control, Tan IIA (2 μM), IR (4 Gy), or a Tan IIA/IR combination. G, *In vivo* tumorigenesis of SCC25-IR cells treated with vehicle control, Tan IIA (2 μM), IR (4 Gy), or a Tan IIA/IR combination. ****p*<0.001. ns, not statistically significant.

.
