# Supplementary material for: Targeting Aurora B kinase with Tanshinone IIA suppresses tumor growth and overcomes radioresistance
Source: Cell Death Dis. 2021 Feb 4;12(2):152. doi: 10.1038/s41419-021-03434-z (PMC7862432; doi:10.1038/s41419-021-03434-z)
Supplement: Supplementary file 2 — Supplementary table [file 41419_2021_3434_MOESM2_ESM.docx]

**Supplementary Table 1. Natural products docking score**

| **Compound** | **Docking_Score** |
| --- | --- |
| Barasertib | -10.80413404 |
| Aloe-emodin | -9.177889974 |
| Rutaecarpine (Rutecarpine) | -8.960516249 |
| Silymarin (Silybin B) | -8.391457476 |
| Chrysin | -8.012669938 |
| Resveratrol | -7.677678141 |
| Aesculin (Esculin) | -7.507279653 |
| Linezolid (Zyvox) | -7.354893646 |
| Taxifolin (Dihydroquercetin) | -7.282683607 |
| Myricetin (Cannabiscetin) | -7.157721412 |
| Genistein | -6.985488768 |
| Cefoperazone (Cefobid) | -6.833994352 |
| Silibinin (Silybin) | -6.766130974 |
| Albendazole (Albenza) | -6.613754009 |
| Asaraldehyde (Asaronaldehyde) | -6.57078513 |
| Adenosine (Adenocard) | -6.496118121 |
| Tanshinone IIA (Tanshinone B) | -6.392340462 |
| Myricitrin (Myricitrine) | -6.38720558 |
| Synephrine HCl | -6.36290282 |
| Orotic acid (6-Carboxyuracil) | -6.337719249 |
| Paeonol (Peonol) | -6.250338821 |
| Reserpine | -6.22475071 |
| Nitrofurazone (Nitrofural) | -6.125911714 |
| Cefdinir (Omnicef) | -5.996720853 |
| Tebipenem pivoxil (L-084) | -5.966005444 |
| Cinchonidine | -5.959607707 |
| L-Adrenaline (Epinephrine) | -5.940374967 |
| Shikimic acid (Shikimate) | -5.890387213 |
| Idebenone | -5.818177796 |
| Natamycin | -5.703497911 |
| Caffeic acid | -5.655238696 |
| 2-Methoxyestradiol | -5.547825385 |
| Artemisinin | -5.440678443 |
| Lincomycin hydrochloride (Lincocin) | -5.426369135 |
| Cloxacillin sodium | -5.361746551 |
| Balofloxacin | -5.323753502 |
| Clindamycin palmitate HCl | -5.30468043 |
| Dopamine hydrochloride (Inotropin) | -5.213444445 |
| Uridine | -5.209689836 |
| Econazole nitrate (Spectazole) | -5.120659299 |
| Fluconazole | -5.103849331 |
| Capsaicin | -4.933890469 |
| **Compound** | **Docking_Score** |
| Dextrose (D-glucose) | -4.877961022 |
| Tetrahydropapaverine hydrochloride | -4.807887281 |
| Artemether | -4.787133451 |
| Oxymatrine (Matrine N-oxide) | -4.763496127 |
| All-trans Retinoic Acid (Tretinoin) | -4.735443088 |
| Bilobalide | -4.627506281 |
| Atropine sulfate monohydrate | -4.613294466 |
| Voriconazole | -4.574395412 |
| Quinine hydrochloride dihydrate | -4.509318716 |
| Forskolin | -4.498808052 |
| Xylose | -4.491073459 |
| Ursodiol (Actigal Urso) | -4.474424312 |
| Ginkgolide A | -4.464655663 |
| Azomycin (2-Nitroimidazole) | -4.402648547 |
| Ginkgolide B | -4.385537935 |
| Oridonin (Isodonol) | -4.382063059 |
| Cortisone acetate (Cortone) | -4.355262321 |
| Sinomenine (Cucoline) | -4.218631565 |
| Gatifloxacin | -4.181529881 |
| Rotenone (Barbasco) | -4.126225495 |
| Gambogic Acid | -4.040074966 |
| 4'-Demethylepipodophyllotoxin | -4.015548985 |
| L-Thyroxine | -3.96501802 |
| Dehydroepiandrosterone (DHEA) | -3.878008703 |
| Sclareolide (Norambreinolide) | -3.845712748 |
| Dexamethasone | -3.796042819 |
| Celastrol | -3.753271792 |
| 5-Aminolevulinic acid hydrochloride | -3.74378468 |
| Artesunate | -3.583384881 |
| Vincristine Sulfate | -3.268541072 |
| Clotrimazole (Canesten) | -3.043940964 |
| Roxithromycin (Roxl-150) | -0.435814057 |
| Clarithromycin (Biaxin Klacid) | -0.532694027 |
