# Supplementary figures and images for: Targeting Aurora B kinase with Tanshinone IIA suppresses tumor growth and overcomes radioresistance
Source: Cell Death Dis. 2021 Feb 4;12(2):152. doi: 10.1038/s41419-021-03434-z (PMC7862432; doi:10.1038/s41419-021-03434-z)

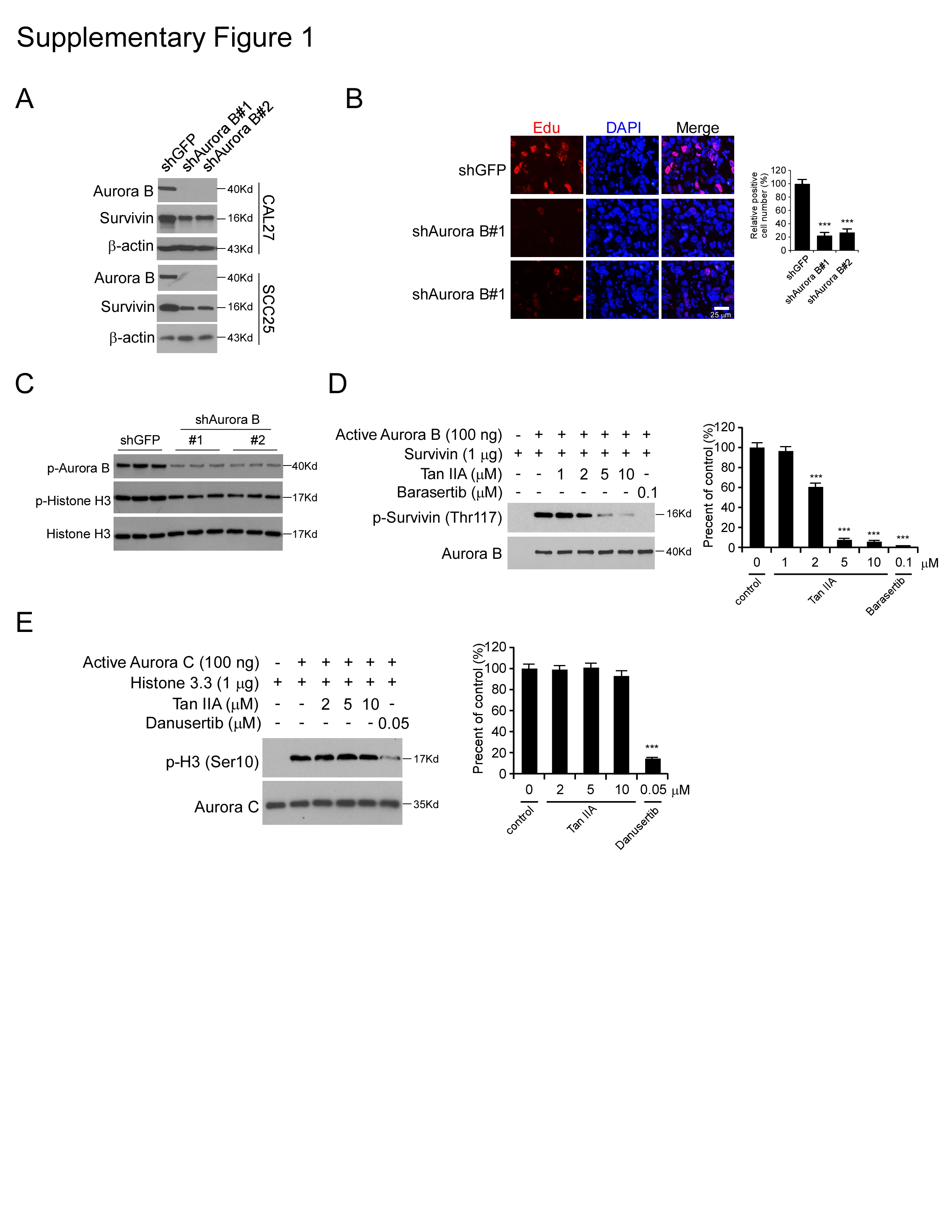

Supplement: Supplementary file 3 — Supplementary figure 1 [file 41419_2021_3434_MOESM3_ESM.jpg]

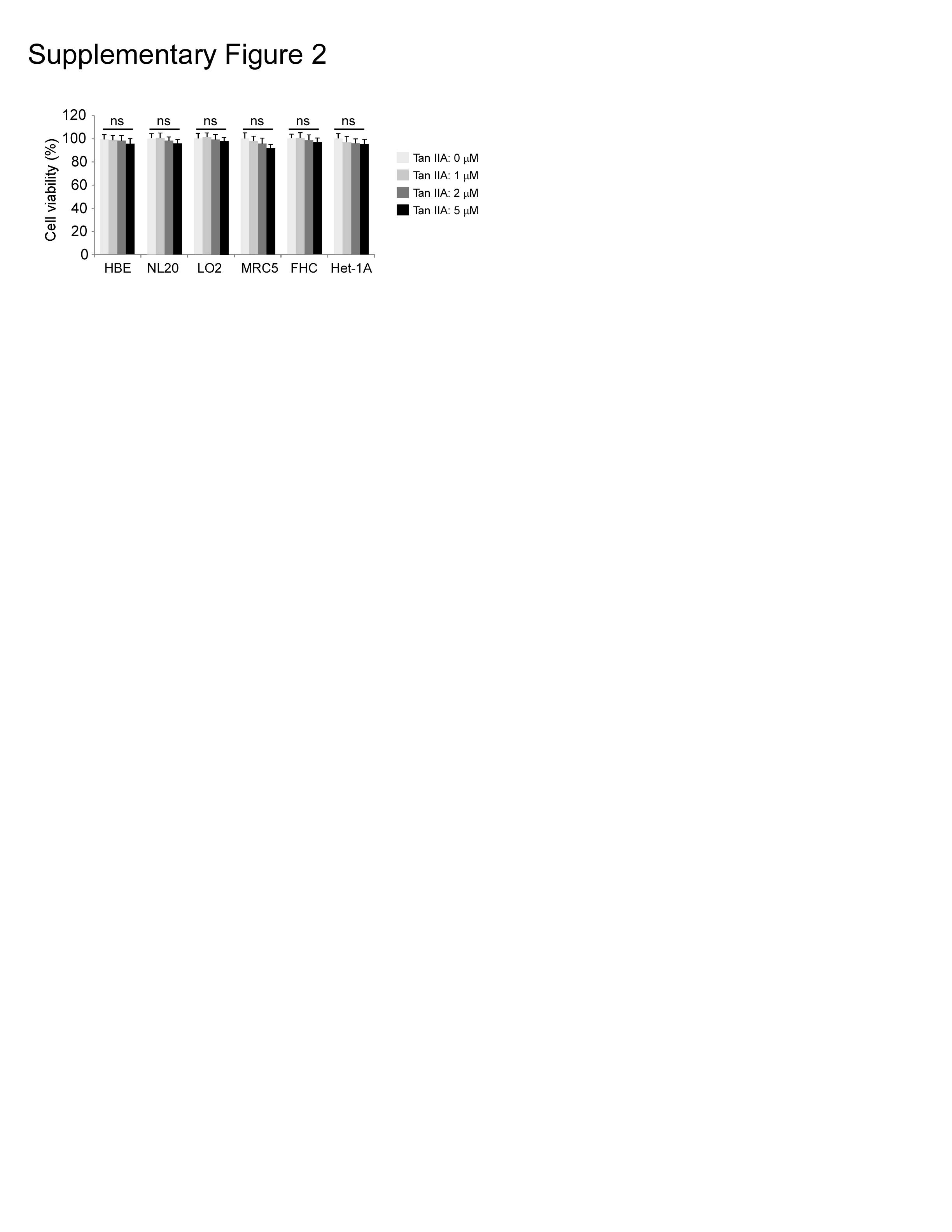

Supplement: Supplementary file 4 — Supplementary figure 2 [file 41419_2021_3434_MOESM4_ESM.jpg]

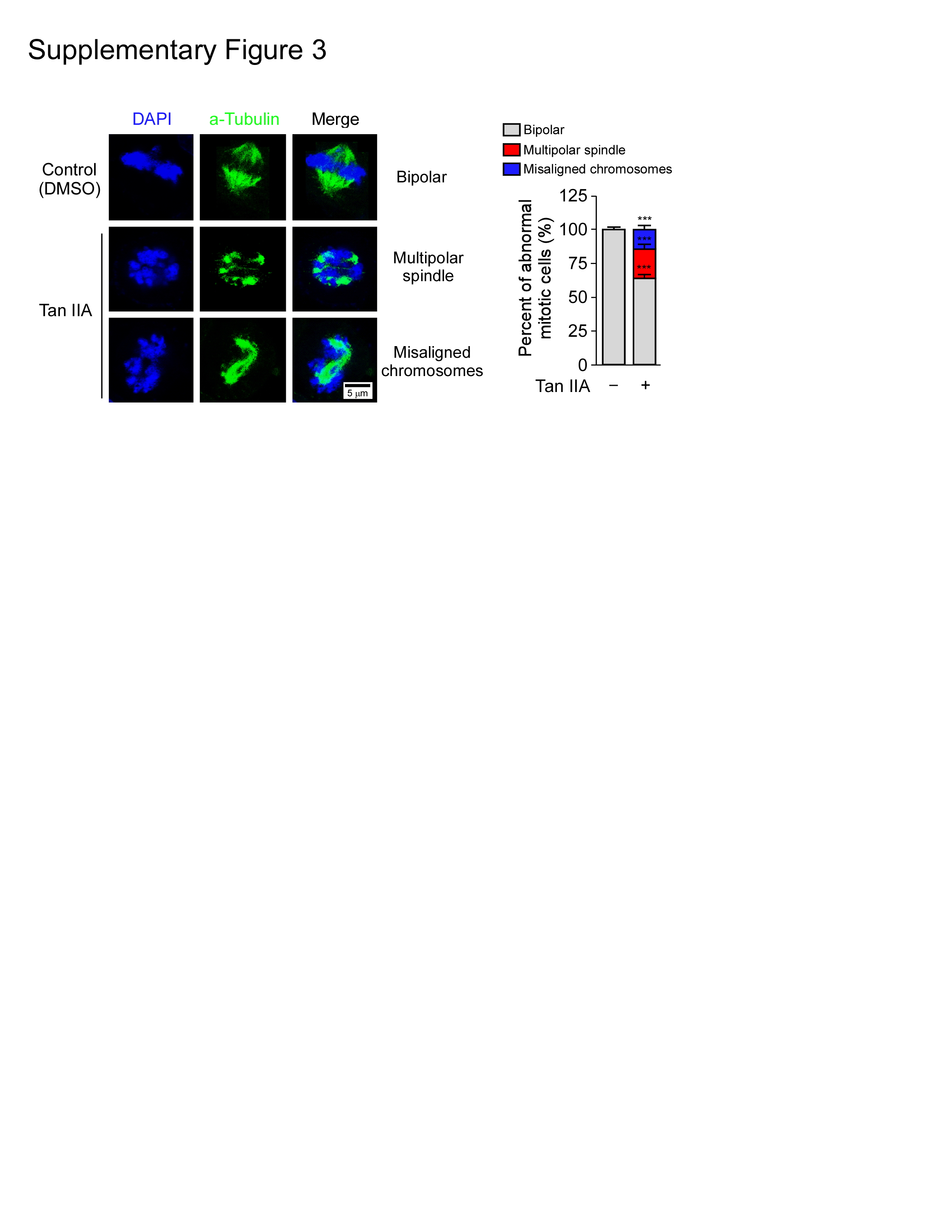

Supplement: Supplementary file 5 — Supplementary figure 3 [file 41419_2021_3434_MOESM5_ESM.jpg]

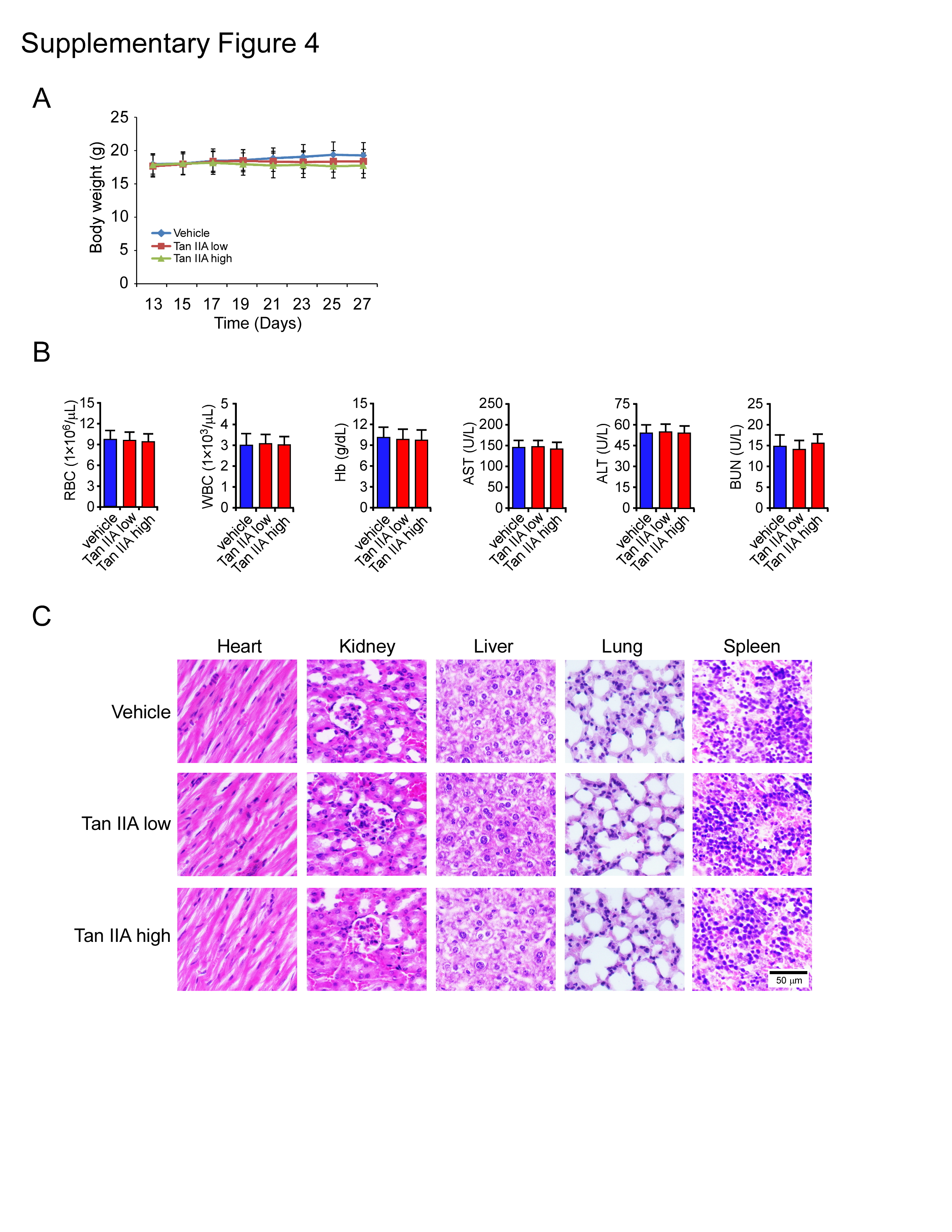

Supplement: Supplementary file 6 — Supplementary figure 4 [file 41419_2021_3434_MOESM6_ESM.jpg]

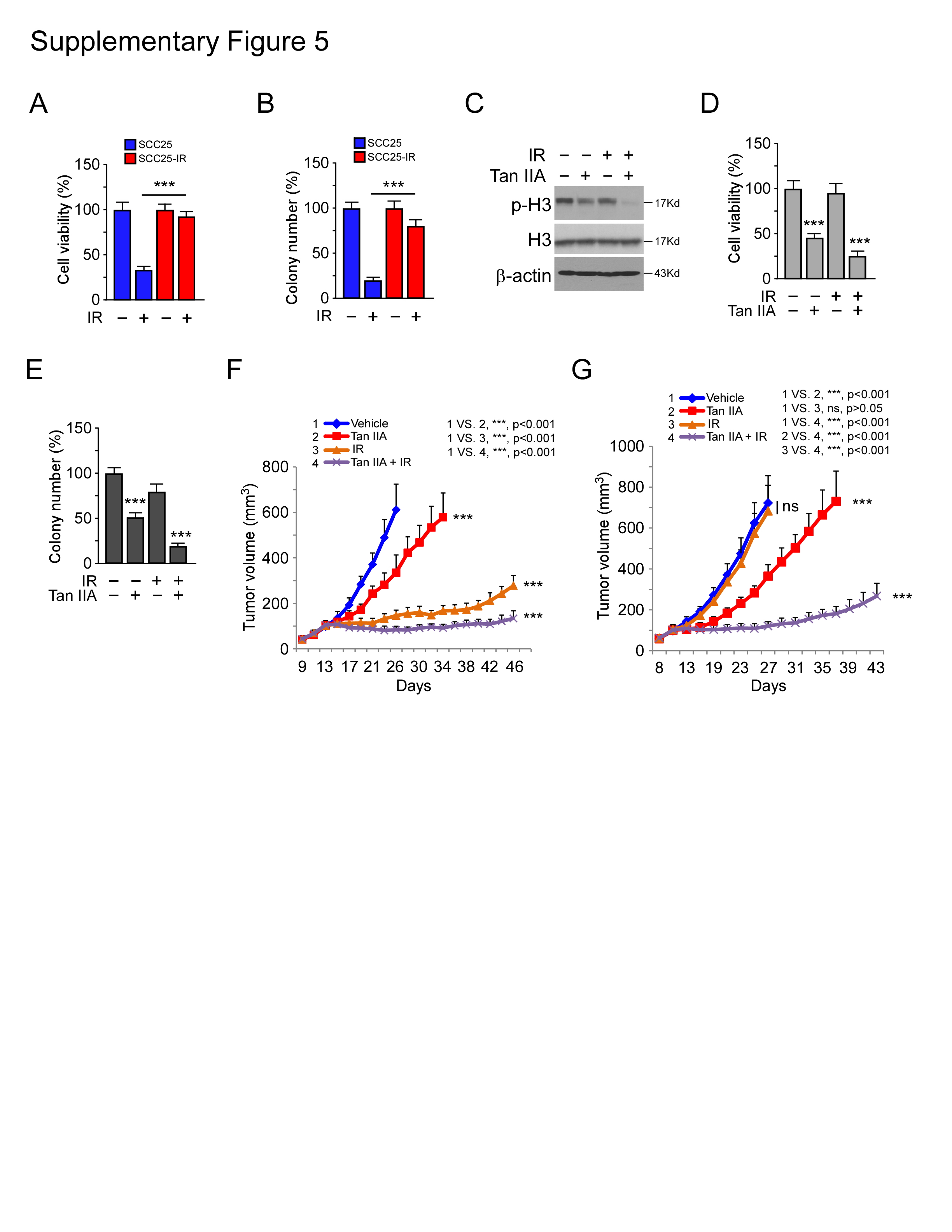

Supplement: Supplementary file 7 — Supplementary figure 5 [file 41419_2021_3434_MOESM7_ESM.jpg]
